# Progress in Bacteriology

**Published:** 1903-09-12

**Authors:** 


### Progress in Bacteriology.

**Serum Therapy.**—Grünbaum[1] summarises some recent observations on serum therapeutics as follows:—Bactericidal sera have met with less success than have antitoxic sera and may be given in too large a dose. Sera should be polyvalent. Tetanus antitoxin must be given early and to obtain the best effect it should be given by intracerebral injection. Letour records five cases successfully treated by this method. Behring suggests that 200 units should be injected in the first 30 hours, and as a prophylactic 10 to 20 units be given. Antistreptococcus serum should always be injected into the subcutaneous tissue and treatment continued after the temperature has fallen. Its effects in cases of puerperal fever have been favourable. The Indian Plague Commission reports that Yersin's is preferable to Lustig's serum, though its curative action is small. Antivenene is a typical antitoxin and is available against the poison of any variety of snake, except Russell's viper. Many successes are reported. With regard to vaccines, Haffkine's antiplague vaccine, when given during incubation, will often abort the disease, and the chances of recovery are increased in an inoculated person attacked by the disease. Wright claims that in the South African campaign the antityphoid vaccination resulted in a reduction of incidence up to 28-fold, a reduction of case mortality of one half, and a diminution of the death-rate amounting to a four-fold reduction. Packard and Willson,[2] reviewing the results of serum treatment, state that since the introduction of antidiphtheritic serum the mortality from all forms of diphtheria has been reduced from 40 per cent. to 15 per cent. The percentage of failures when used for prophylactic purposes has varied from 1 to 6 per cent., and so great has been the success that in many children's hospitals preventive injections are given to every child admitted. Antidiphtheritic serum has also been used in the treatment of lobar pneumonia and whooping cough. Except in Italy where the tetanus is apparently a milder disease the results of treatment with tetanus antitoxin have been disappointing, the mortality still ranging from 42 to 64·7 per cent. Of 52 cases of vaccine tetanus with a general mortality of 78·8 per cent., 39 treated without antitoxin showed a mortality of 82 per cent.; 13 treated with antitoxin, a mortality of 76·9 per cent. Subcutaneous injections can only affect the poison en route from the primary wound to the nervous centres, and once symptoms have appeared, intra-cerebral injections afford the best help. In pre-antitoxin days the mortality amongst acute cases was from 70 to 90 per cent., and amongst chronic cases 20 to 40 per cent. Jacobi recommends that five to ten cubic centimetres of cerebro-spinal fluid be withdrawn by lumbar puncture, and that ten to twenty cubic centimetres of antitoxin be injected into the spinal canal very slowly and under weak pressure. The writers agree that Chantemesse's experiments prove that a curative anti-typhoid serum can be prepared, and results seem sufficiently encouraging to warrant an extended use of such a preparation. Treatment by antistreptococcic serum has not been as successful as anticipated owing to the fact that few of the sera are polyvalent. It is worthy of trial in pernicious and simple anæmia. Yersin's plague serum has yielded good results, and is best administered in doses of 20 cubic centimetres intravenously. Fisch and Maragliano have produced antitubercle sera, which are useful adjuvants in the control of tuberculosis, and which if used early may result in cure, and even when used late may tend to retard the process and promote healing. With regard to antivenene the writers state that it is least potent against the bite of the rattlesnake and viper. The dose is 20 to 40 cubic centimetres. Sera have also been prepared against dysentery (Shiga), yellow fever (Terni), anthrax (Liscia), leprosy (Carasquilla), glanders and syphilis (Moore).

**The Chancroid Bacillus.**—Ducrey first described a specific bacillus of soft chancre, and his observation has since been confirmed by many observers. The organism is a bacillus about $1{\cdot}5\mu \times {\cdot}5\mu$, with polar staining, and is found in the pus of chancroidal ulcerations and buboes. The bacilli lie within or without the cells in long chains, stained with aniline dyes, but are decolorised by Gram's method. Coagulated rabbit's-blood serum was the best culture medium, and the disease had been reproduced by inoculation upon monkeys. Davis[3] has found this organism in 32 out of 40 cases in pure or mixed cultures. The media used were rabbit's-blood serum uncoagulated, rabbit's-blood bouillon, and freshly drawn human blood. In fluid media the chancroid bacilli form a flocculent precipitate consisting of long undivided filaments; on solid media the colonies are small, round, and greyish, and the organism quickly undergoes involution. Inoculation upon a monkey of the genus Macacus nemestrinus resulted in the formation of shallow ulcers, from which the bacillus could again be recovered. Davis notes that the cultivation of the same organism in a pure state from lesions on the hands, clinically resembling chancroid, in the absence of genital lesions, indicates that chancroid may be primary on an extragenital site.

**Tubercle Bacilli in Lymphoid Tissue.**—Macfadyen and Macconkey[4] have made a post-mortem examination of the mesenteric glands and other tissues of children dying from tuberculosis and other diseases. The tissues immediately on removal were placed in a mixture of equal parts of glycerine and normal saline triturated and disintegrated until a pulpy mass was obtained suitable for injection by means of a hypodermic needle. Guinea-pigs were the animals used, and one-half of the mass was injected subcutaneously, and the remainder into the peritoneal cavity. All the animals surviving after six or eight weeks were killed and examined. Twenty-eight cases were examined, and all the patients were under eight years of age. In eight cases the cause of death was stated to be tuberculosis. Virulent tubercle bacilli were present in the mesenteric glands in ten of the 28 cases. In the eight tuberculous cases tubercle bacilli were found in five, although there was no intestinal lesion. Of the other glands, tubercle bacilli were found in the bronchial glands in one case out of four, viz., a case of general tuberculosis. The tubercle bacillus was detected in the mesenteric glands of a stillborn infant. The excised tissues were examined in 44 cases of adenoids and 34 cases of enlarged tonsils in young children. Inoculation experiments were negative in all. A parallel series of observations by microscopic examination only showed the vast superiority of the inoculation method.

**Widal's Reaction and Weil's Disease.**—Eckardt[5] records a case of Weil's disease in which a well-marked Widal's reaction was obtained. Weil's disease is an infectious disorder characterised by fever, enlargement of the liver and spleen, hæmorrhagic nephritis, epistaxis, jaundice, and pains in the limbs. Jäger attributed the disease to infection by B. coli, and Banti to the B. icterogenes capsulatus. In the case under notice the disease had been contracted from a person sharing the same room and in all points the case was clinically one of Weil's disease. Widal's reaction was obtained on the tenth day with dilution 1 : 10, 1 : 50, 1 : 100 in fifteen minutes and 1 : 1000 in two hours. Subsequent examinations of the blood confirmed this observation. The blood of the first sufferer was also examined and although the patient had been convalescent for some days a complete reaction was obtained. Whilst it is possible that these were cases of abortive typhoid there are several points against this theory. Icterus in enteric fever is a rare complication only occurring, according to Liebermeister, in 26 out of 1,420 cases. Grünbaum has recorded that although bile has no agglutinating action on cultures of typhoid bacilli yet the blood of persons suffering from jaundice, altogether apart from enteric fever, has marked agglutinating properties, especially if the jaundice be recent and intense. A further investigation of the bacteriology of Weil's disease may possibly show that it is dependent on the life of growth of the B. typhosus.

**Bacteria and High-frequency Currents.**—Foulerton and Kellas,[6] who formerly demonstrated the bactericidal action of electrical currents of high potential and rapid frequency, now see cause to modify this view. They have discovered that in the presence of air the currents form free nitrous and nitric acids, that these are absorbed by the water containing the bacteria, and the acid solution thus formed kills the organisms. When the experiments were repeated in an atmosphere of hydrogen the bacteria survived the highest discharges for at least an hour and a half, so that the effect can be but slight. Possibly the beneficial effects of high-frequency currents on ulcerative processes are due to nascent and active nitrogen compounds formed from the air and carried to and penetrating the tissues in minute quantities.

[1] Practitioner, Nov. 9, 1902. [2] Amer. Jour. Med. Sci., Dec. 9, 1902. [3] Jour. Med. Rec., June 9, 1903. [4] Brit. Med. Jour., July 18, 1903. [5] Munch. Med. Woch., Vol. XLIX., 1902. [6] Brit. Med. Jour., April 25, 1903.

